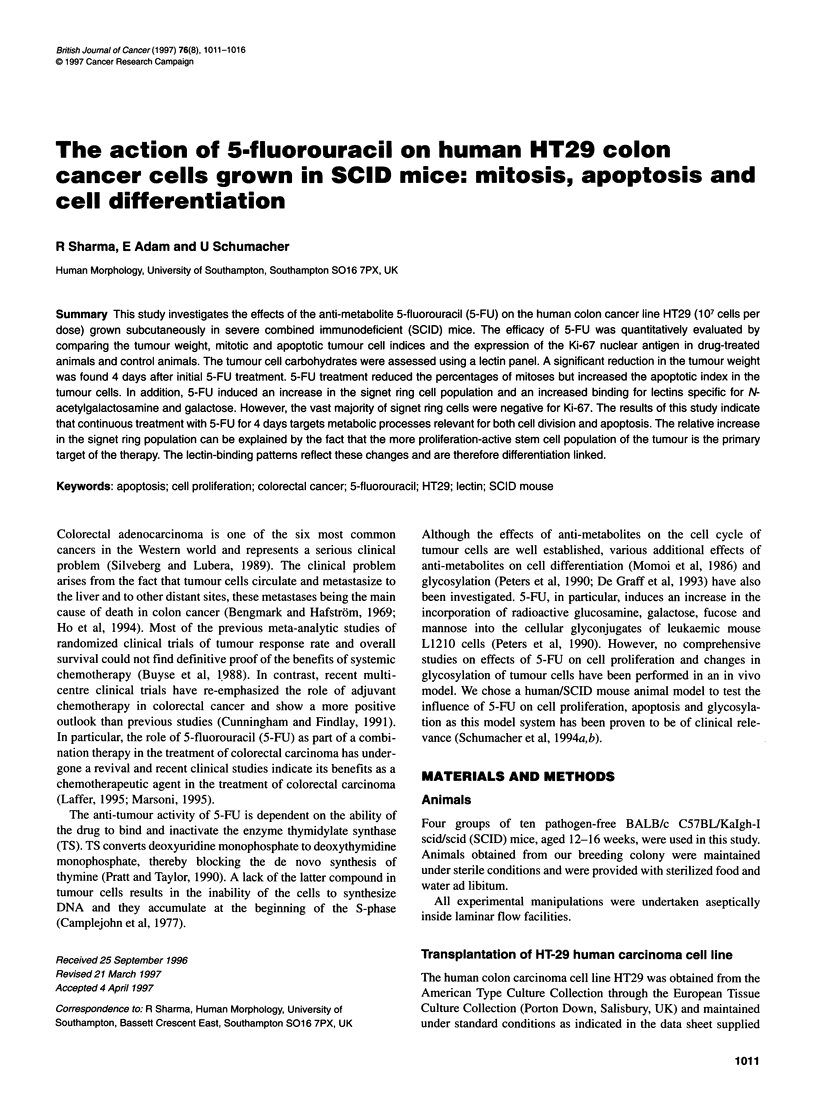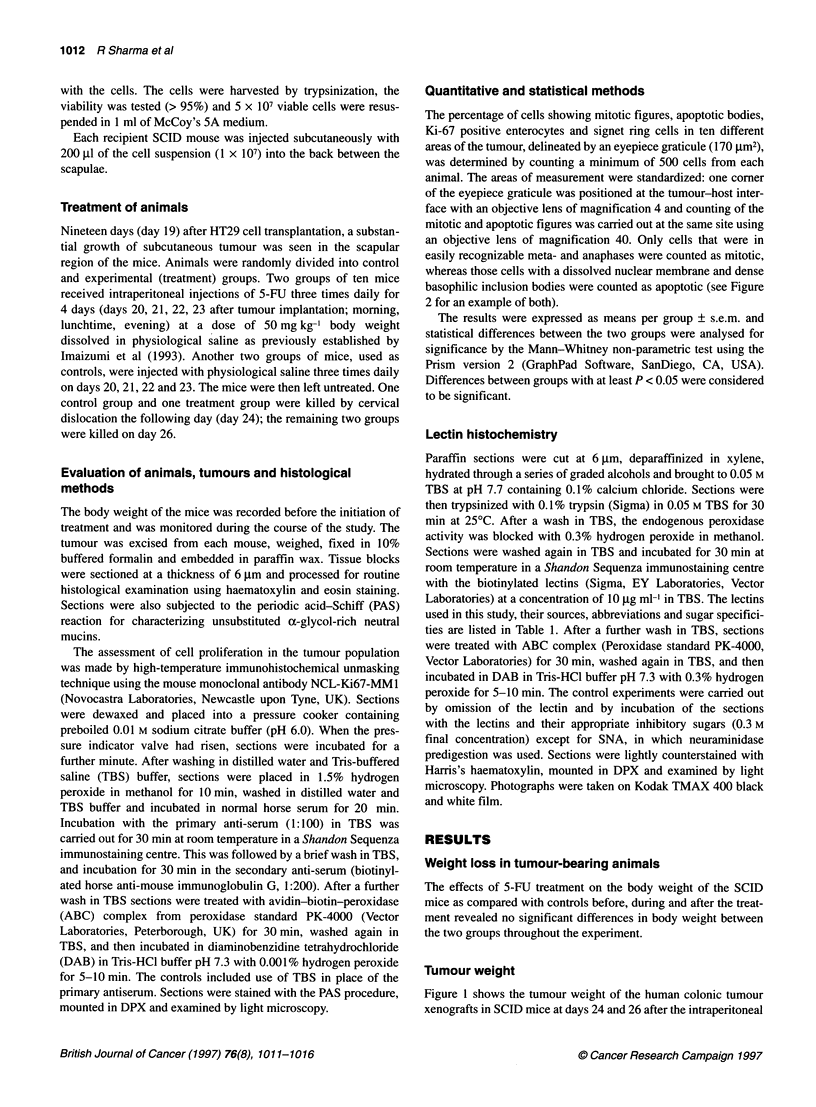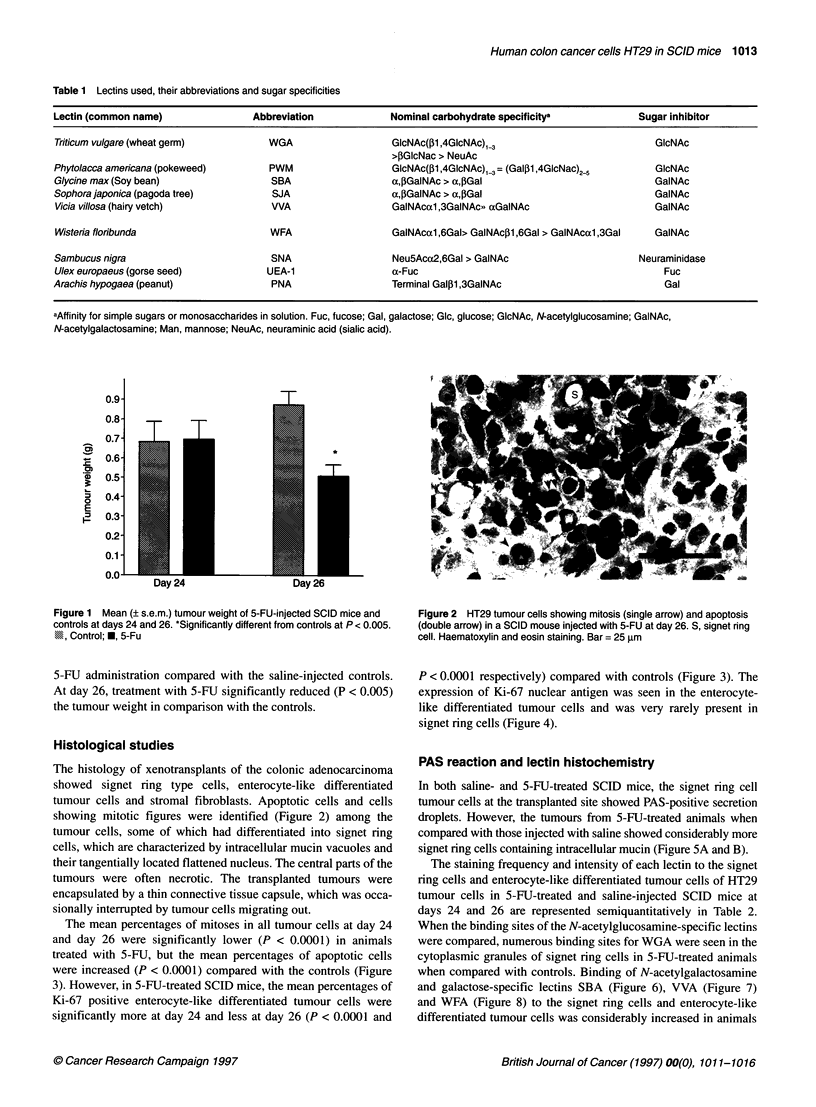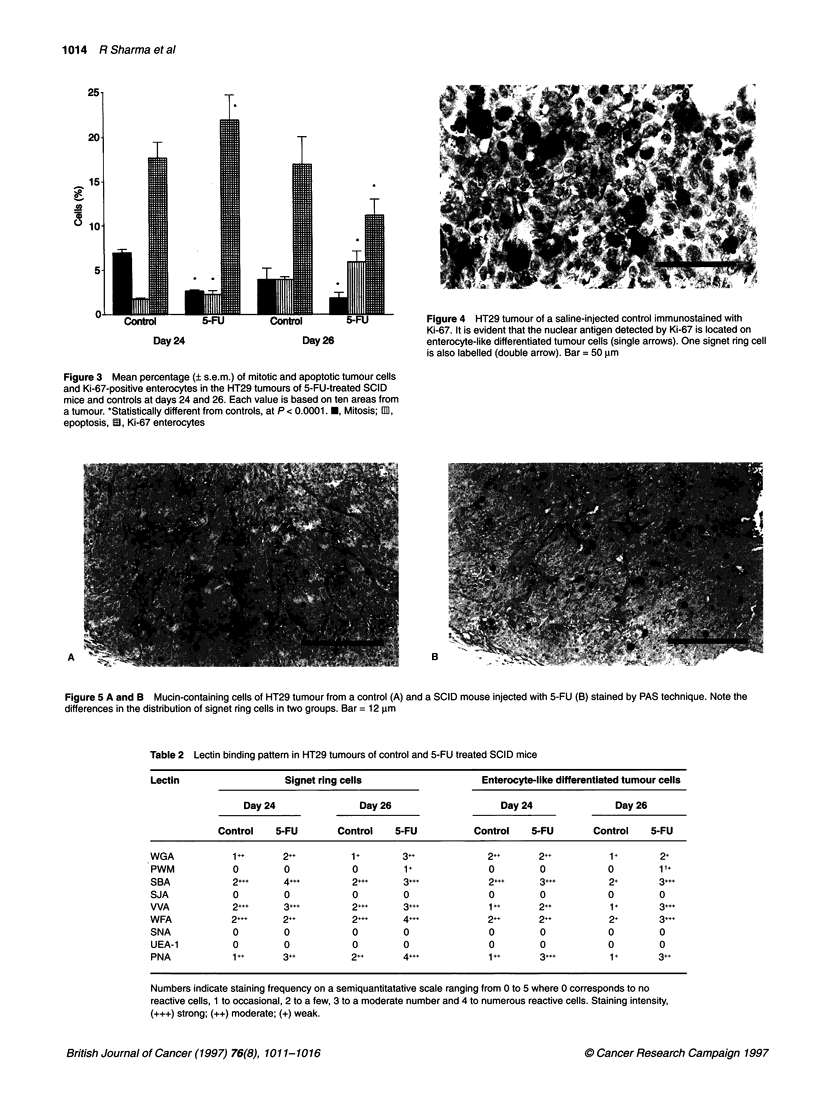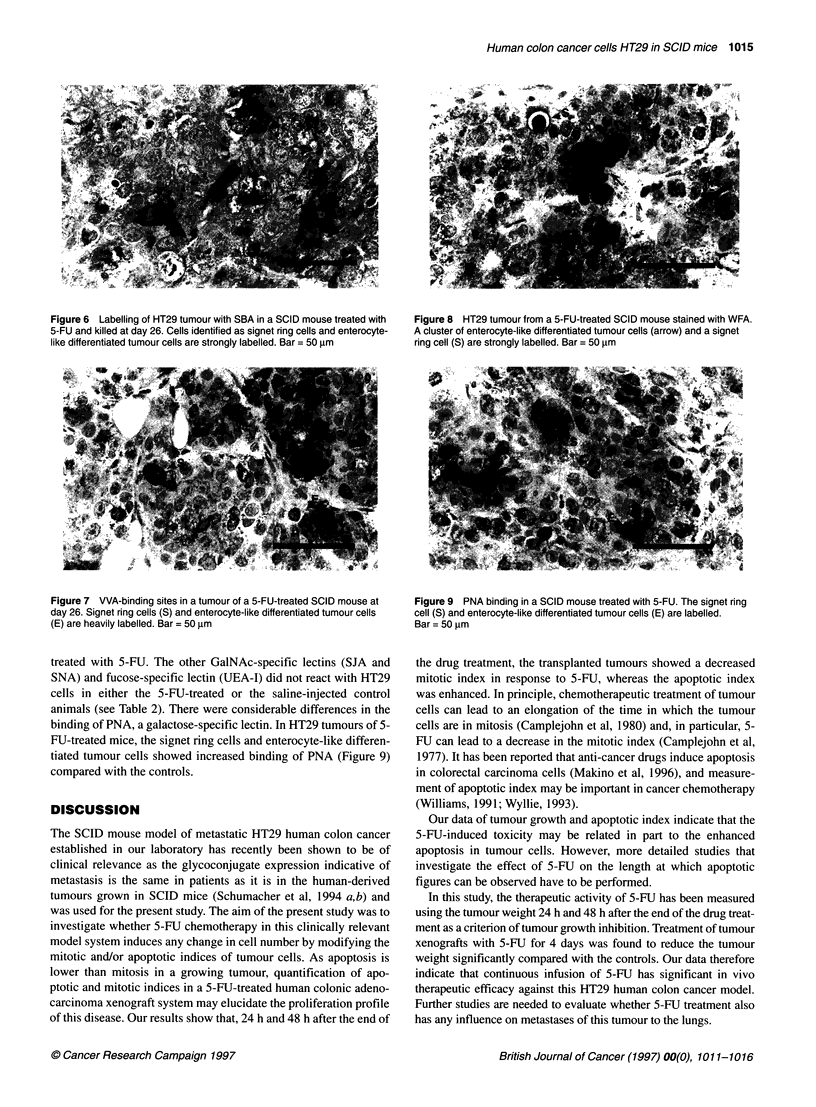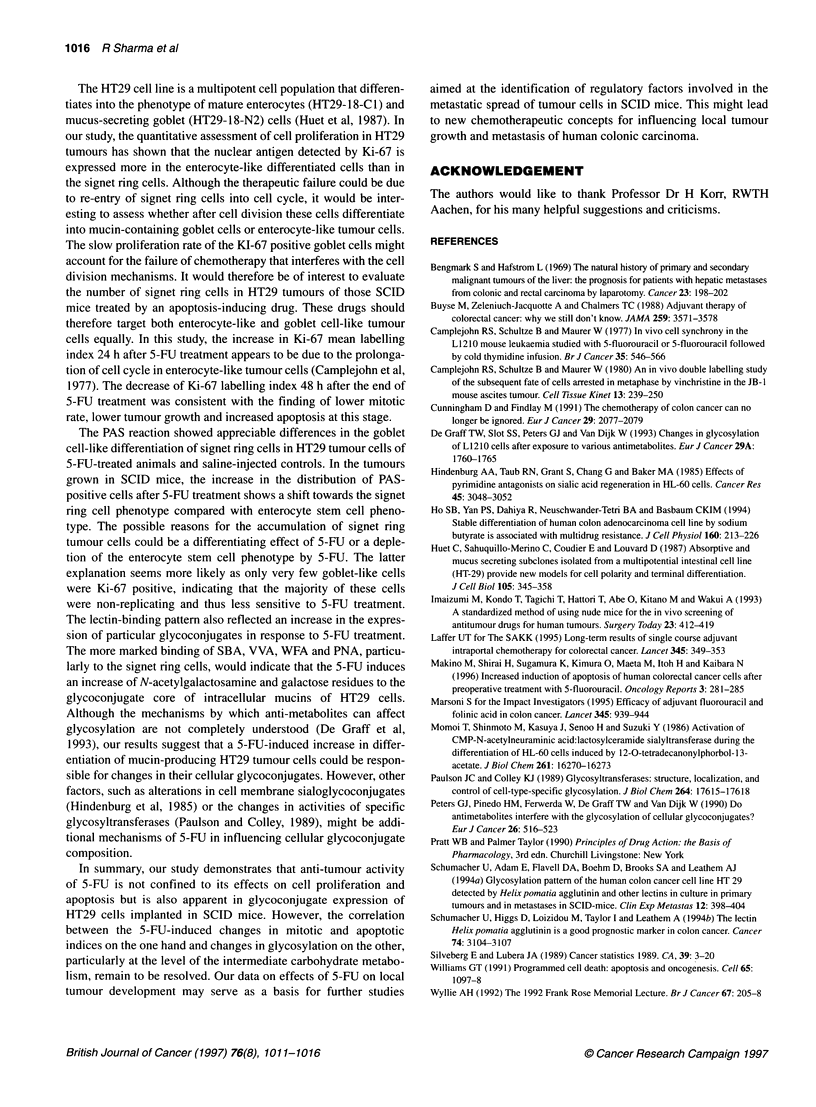# The action of 5-fluorouracil on human HT29 colon cancer cells grown in SCID mice: mitosis, apoptosis and cell differentiation.

**DOI:** 10.1038/bjc.1997.500

**Published:** 1997

**Authors:** R. Sharma, E. Adam, U. Schumacher

**Affiliations:** Human Morphology, University of Southampton, UK.

## Abstract

**Images:**


British Journal of Cancer (1997) 76(8), 1011–1016
© 1997 Cancer Research Campaign

### The action of 5-fluorouracil on human HT29 colon cancer cells grown in SCID mice: mitosis, apoptosis and cell differentiation

**R Sharma, E Adam and U Schumacher**

Human Morphology, University of Southampton, Southampton SO16 7PX, UK

**Summary** This study investigates the effects of the anti-metabolite 5-fluorouracil (5-FU) on the human colon cancer line HT29 ($10^7$ cells per dose) grown subcutaneously in severe combined immunodeficient (SCID) mice. The efficacy of 5-FU was quantitatively evaluated by comparing the tumour weight, mitotic and apoptotic tumour cell indices and the expression of the Ki-67 nuclear antigen in drug-treated animals and control animals. The tumour cell carbohydrates were assessed using a lectin panel. A significant reduction in the tumour weight was found 4 days after initial 5-FU treatment. 5-FU treatment reduced the percentages of mitoses but increased the apoptotic index in the tumour cells. In addition, 5-FU induced an increase in the signet ring cell population and an increased binding for lectins specific for *N*-acetylgalactosamine and galactose. However, the vast majority of signet ring cells were negative for Ki-67. The results of this study indicate that continuous treatment with 5-FU for 4 days targets metabolic processes relevant for both cell division and apoptosis. The relative increase in the signet ring population can be explained by the fact that the more proliferation-active stem cell population of the tumour is the primary target of the therapy. The lectin-binding patterns reflect these changes and are therefore differentiation linked.

**Keywords**: apoptosis; cell proliferation; colorectal cancer; 5-fluorouracil; HT29; lectin; SCID mouse

Colorectal adenocarcinoma is one of the six most common cancers in the Western world and represents a serious clinical problem (Silveberg and Lubera, 1989). The clinical problem arises from the fact that tumour cells circulate and metastasize to the liver and to other distant sites, these metastases being the main cause of death in colon cancer (Bengmark and Hafström, 1969; Ho et al, 1994). Most of the previous meta-analytic studies of randomized clinical trials of tumour response rate and overall survival could not find definitive proof of the benefits of systemic chemotherapy (Buyse et al, 1988). In contrast, recent multi-centre clinical trials have re-emphasized the role of adjuvant chemotherapy in colorectal cancer and show a more positive outlook than previous studies (Cunningham and Findlay, 1991). In particular, the role of 5-fluorouracil (5-FU) as part of a combination therapy in the treatment of colorectal carcinoma has undergone a revival and recent clinical studies indicate its benefits as a chemotherapeutic agent in the treatment of colorectal carcinoma (Laffer, 1995; Marsoni, 1995).

The anti-tumour activity of 5-FU is dependent on the ability of the drug to bind and inactivate the enzyme thymidylate synthase (TS). TS converts deoxyuridine monophosphate to deoxythymidine monophosphate, thereby blocking the de novo synthesis of thymine (Pratt and Taylor, 1990). A lack of the latter compound in tumour cells results in the inability of the cells to synthesize DNA and they accumulate at the beginning of the S-phase (Camplejohn et al, 1977).

*Received 25 September 1996*
*Revised 21 March 1997*
*Accepted 4 April 1997*

*Correspondence to:* R Sharma, Human Morphology, University of Southampton, Bassett Crescent East, Southampton SO16 7PX, UK

Although the effects of anti-metabolites on the cell cycle of tumour cells are well established, various additional effects of anti-metabolites on cell differentiation (Momoi et al, 1986) and glycosylation (Peters et al, 1990; De Graff et al, 1993) have also been investigated. 5-FU, in particular, induces an increase in the incorporation of radioactive glucosamine, galactose, fucose and mannose into the cellular glyconjugates of leukaemic mouse L1210 cells (Peters et al, 1990). However, no comprehensive studies on effects of 5-FU on cell proliferation and changes in glycosylation of tumour cells have been performed in an in vivo model. We chose a human/SCID mouse animal model to test the influence of 5-FU on cell proliferation, apoptosis and glycosylation as this model system has been proven to be of clinical relevance (Schumacher et al, 1994*a*,*b*).

### MATERIALS AND METHODS

### Animals

Four groups of ten pathogen-free BALB/c C57BL/Kalgh-I scid/scid (SCID) mice, aged 12–16 weeks, were used in this study. Animals obtained from our breeding colony were maintained under sterile conditions and were provided with sterilized food and water ad libitum.

All experimental manipulations were undertaken aseptically inside laminar flow facilities.

### Transplantation of HT-29 human carcinoma cell line

The human colon carcinoma cell line HT29 was obtained from the American Type Culture Collection through the European Tissue Culture Collection (Porton Down, Salisbury, UK) and maintained under standard conditions as indicated in the data sheet supplied with the cells. The cells were harvested by trypsinization, the viability was tested (> 95%) and $5 \times 10^7$ viable cells were resuspended in 1 ml of McCoy's 5A medium.

Each recipient SCID mouse was injected subcutaneously with 200 μl of the cell suspension ($1 \times 10^7$) into the back between the scapulae.

### Treatment of animals

Nineteen days (day 19) after HT29 cell transplantation, a substantial growth of subcutaneous tumour was seen in the scapular region of the mice. Animals were randomly divided into control and experimental (treatment) groups. Two groups of ten mice received intraperitoneal injections of 5-FU three times daily for 4 days (days 20, 21, 22, 23 after tumour implantation; morning, lunchtime, evening) at a dose of 50 mg $kg^{-1}$ body weight dissolved in physiological saline as previously established by Imaizumi et al (1993). Another two groups of mice, used as controls, were injected with physiological saline three times daily on days 20, 21, 22 and 23. The mice were then left untreated. One control group and one treatment group were killed by cervical dislocation the following day (day 24); the remaining two groups were killed on day 26.

### Evaluation of animals, tumours and histological methods

The body weight of the mice was recorded before the initiation of treatment and was monitored during the course of the study. The tumour was excised from each mouse, weighed, fixed in 10% buffered formalin and embedded in paraffin wax. Tissue blocks were sectioned at a thickness of 6 μm and processed for routine histological examination using haematoxylin and eosin staining. Sections were also subjected to the periodic acid–Schiff (PAS) reaction for characterizing unsubstituted α-glycol-rich neutral mucins.

The assessment of cell proliferation in the tumour population was made by high-temperature immunohistochemical unmasking technique using the mouse monoclonal antibody NCL-Ki67-MM1 (Novocastra Laboratories, Newcastle upon Tyne, UK). Sections were dewaxed and placed into a pressure cooker containing preboiled 0.01 M sodium citrate buffer (pH 6.0). When the pressure indicator valve had risen, sections were incubated for a further minute. After washing in distilled water and Tris-buffered saline (TBS) buffer, sections were placed in 1.5% hydrogen peroxide in methanol for 10 min, washed in distilled water and TBS buffer and incubated in normal horse serum for 20 min. Incubation with the primary anti-serum (1:100) in TBS was carried out for 30 min at room temperature in a *Shandon* Sequenza immunostaining centre. This was followed by a brief wash in TBS, and incubation for 30 min in the secondary anti-serum (biotinylated horse anti-mouse immunoglobulin G, 1:200). After a further wash in TBS sections were treated with avidin–biotin–peroxidase (ABC) complex from peroxidase standard PK-4000 (Vector Laboratories, Peterborough, UK) for 30 min, washed again in TBS, and then incubated in diaminobenzidine tetrahydrochloride (DAB) in Tris-HCl buffer pH 7.3 with 0.001% hydrogen peroxide for 5–10 min. The controls included use of TBS in place of the primary antiserum. Sections were stained with the PAS procedure, mounted in DPX and examined by light microscopy.

### Quantitative and statistical methods

The percentage of cells showing mitotic figures, apoptotic bodies, Ki-67 positive enterocytes and signet ring cells in ten different areas of the tumour, delineated by an eyepiece graticule (170 $μm^2$), was determined by counting a minimum of 500 cells from each animal. The areas of measurement were standardized: one corner of the eyepiece graticule was positioned at the tumour–host interface with an objective lens of magnification 4 and counting of the mitotic and apoptotic figures was carried out at the same site using an objective lens of magnification 40. Only cells that were in easily recognizable meta- and anaphases were counted as mitotic, whereas those cells with a dissolved nuclear membrane and dense basophilic inclusion bodies were counted as apoptotic (see Figure 2 for an example of both).

The results were expressed as means per group ± s.e.m. and statistical differences between the two groups were analysed for significance by the Mann–Whitney non-parametric test using the Prism version 2 (GraphPad Software, SanDiego, CA, USA). Differences between groups with at least $P < 0.05$ were considered to be significant.

### Lectin histochemistry

Paraffin sections were cut at 6 μm, deparaffinized in xylene, hydrated through a series of graded alcohols and brought to 0.05 M TBS at pH 7.7 containing 0.1% calcium chloride. Sections were then trypsinized with 0.1% trypsin (Sigma) in 0.05 M TBS for 30 min at 25°C. After a wash in TBS, the endogenous peroxidase activity was blocked with 0.3% hydrogen peroxide in methanol. Sections were washed again in TBS and incubated for 30 min at room temperature in a *Shandon* Sequenza immunostaining centre with the biotinylated lectins (Sigma, EY Laboratories, Vector Laboratories) at a concentration of 10 μg $ml^{-1}$ in TBS. The lectins used in this study, their sources, abbreviations and sugar specificities are listed in Table 1. After a further wash in TBS, sections were treated with ABC complex (Peroxidase standard PK-4000, Vector Laboratories) for 30 min, washed again in TBS, and then incubated in DAB in Tris-HCl buffer pH 7.3 with 0.3% hydrogen peroxide for 5–10 min. The control experiments were carried out by omission of the lectin and by incubation of the sections with the lectins and their appropriate inhibitory sugars (0.3 M final concentration) except for SNA, in which neuraminidase predigestion was used. Sections were lightly counterstained with Harris's haematoxylin, mounted in DPX and examined by light microscopy. Photographs were taken on Kodak TMAX 400 black and white film.

### RESULTS

### Weight loss in tumour-bearing animals

The effects of 5-FU treatment on the body weight of the SCID mice as compared with controls before, during and after the treatment revealed no significant differences in body weight between the two groups throughout the experiment.

### Tumour weight

Figure 1 shows the tumour weight of the human colonic tumour xenografts in SCID mice at days 24 and 26 after the intraperitoneal

 © Cancer Research Campaign 1997

**Table 1** Lectins used, their abbreviations and sugar specificities

| Lectin (common name) | Abbreviation | Nominal carbohydrate specificity[a] | Sugar inhibitor |
|---|---|---|---|
| *Triticum vulgare* (wheat germ) | WGA | GlcNAc(β1,4GlcNAc)$_{1-3}$ >βGlcNac > NeuAc | GlcNAc |
| *Phytolacca americana* (pokeweed) | PWM | GlcNAc(β1,4GlcNAc)$_{1-3}$ = (Galβ1,4GlcNac)$_{2-5}$ | GlcNAc |
| *Glycine max* (Soy bean) | SBA | α,βGalNAc > α,βGal | GalNAc |
| *Sophora japonica* (pagoda tree) | SJA | α,βGalNAc > α,βGal | GalNAc |
| *Vicia villosa* (hairy vetch) | VVA | GalNAcα1,3GalNAc» αGalNAc | GalNAc |
| *Wisteria floribunda* | WFA | GalNAcα1,6Gal> GalNAcβ1,6Gal > GalNAcα1,3Gal | GalNAc |
| *Sambucus nigra* | SNA | Neu5Acα2,6Gal > GalNAc | Neuraminidase |
| *Ulex europaeus* (gorse seed) | UEA-1 | α-Fuc | Fuc |
| *Arachis hypogaea* (peanut) | PNA | Terminal Galβ1,3GalNAc | Gal |

[a]Affinity for simple sugars or monosaccharides in solution. Fuc, fucose; Gal, galactose; Glc, glucose; GlcNAc, *N*-acetylglucosamine; GalNAc, *N*-acetylgalactosamine; Man, mannose; NeuAc, neuraminic acid (sialic acid).

**Figure 1** Mean (± s.e.m.) tumour weight of 5-FU-injected SCID mice and controls at days 24 and 26. *Significantly different from controls at $P < 0.005$. ▨, Control; ■, 5-Fu

**Figure 2** HT29 tumour cells showing mitosis (single arrow) and apoptosis (double arrow) in a SCID mouse injected with 5-FU at day 26. S, signet ring cell. Haematoxylin and eosin staining. Bar = 25 μm

5-FU administration compared with the saline-injected controls. At day 26, treatment with 5-FU significantly reduced (P < 0.005) the tumour weight in comparison with the controls.

### Histological studies

The histology of xenotransplants of the colonic adenocarcinoma showed signet ring type cells, enterocyte-like differentiated tumour cells and stromal fibroblasts. Apoptotic cells and cells showing mitotic figures were identified (Figure 2) among the tumour cells, some of which had differentiated into signet ring cells, which are characterized by intracellular mucin vacuoles and their tangentially located flattened nucleus. The central parts of the tumours were often necrotic. The transplanted tumours were encapsulated by a thin connective tissue capsule, which was occasionally interrupted by tumour cells migrating out.

The mean percentages of mitoses in all tumour cells at day 24 and day 26 were significantly lower ($P < 0.0001$) in animals treated with 5-FU, but the mean percentages of apoptotic cells were increased ($P < 0.0001$) compared with the controls (Figure 3). However, in 5-FU-treated SCID mice, the mean percentages of Ki-67 positive enterocyte-like differentiated tumour cells were significantly more at day 24 and less at day 26 ($P < 0.0001$ and $P < 0.0001$ respectively) compared with controls (Figure 3). The expression of Ki-67 nuclear antigen was seen in the enterocyte-like differentiated tumour cells and was very rarely present in signet ring cells (Figure 4).

### PAS reaction and lectin histochemistry

In both saline- and 5-FU-treated SCID mice, the signet ring cell tumour cells at the transplanted site showed PAS-positive secretion droplets. However, the tumours from 5-FU-treated animals when compared with those injected with saline showed considerably more signet ring cells containing intracellular mucin (Figure 5A and B).

The staining frequency and intensity of each lectin to the signet ring cells and enterocyte-like differentiated tumour cells of HT29 tumour cells in 5-FU-treated and saline-injected SCID mice at days 24 and 26 are represented semiquantitatively in Table 2. When the binding sites of the *N*-acetylglucosamine-specific lectins were compared, numerous binding sites for WGA were seen in the cytoplasmic granules of signet ring cells in 5-FU-treated animals when compared with controls. Binding of *N*-acetylgalactosamine and galactose-specific lectins SBA (Figure 6), VVA (Figure 7) and WFA (Figure 8) to the signet ring cells and enterocyte-like differentiated tumour cells was considerably increased in animals

© Cancer Research Campaign 1997

**Figure 3** Mean percentage (± s.e.m.) of mitotic and apoptotic tumour cells and Ki-67-positive enterocytes in the HT29 tumours of 5-FU-treated SCID mice and controls at days 24 and 26. Each value is based on ten areas from a tumour. *Statistically different from controls, at $P < 0.0001$. ■, Mitosis; ▥, epoptosis, ▦, Ki-67 enterocytes

**Figure 4** HT29 tumour of a saline-injected control immunostained with Ki-67. It is evident that the nuclear antigen detected by Ki-67 is located on enterocyte-like differentiated tumour cells (single arrows). One signet ring cell is also labelled (double arrow). Bar = 50 μm

**Figure 5 A and B** Mucin-containing cells of HT29 tumour from a control (A) and a SCID mouse injected with 5-FU (B) stained by PAS technique. Note the differences in the distribution of signet ring cells in two groups. Bar = 12 μm

**Table 2** Lectin binding pattern in HT29 tumours of control and 5-FU treated SCID mice

| Lectin | Signet ring cells | | | | Enterocyte-like differentiated tumour cells | | | |
|---|---|---|---|---|---|---|---|---|
| | Day 24 | | Day 26 | | Day 24 | | Day 26 | |
| | Control | 5-FU | Control | 5-FU | Control | 5-FU | Control | 5-FU |
| WGA | $1^{++}$ | $2^{++}$ | $1^{+}$ | $3^{++}$ | $2^{++}$ | $2^{++}$ | $1^{+}$ | $2^{+}$ |
| PWM | 0 | 0 | 0 | $1^{+}$ | 0 | 0 | 0 | $1^{1+}$ |
| SBA | $2^{+++}$ | $4^{+++}$ | $2^{+++}$ | $3^{+++}$ | $2^{+++}$ | $3^{+++}$ | $2^{+}$ | $3^{+++}$ |
| SJA | 0 | 0 | 0 | 0 | 0 | 0 | 0 | 0 |
| VVA | $2^{+++}$ | $3^{+++}$ | $2^{+++}$ | $3^{+++}$ | $1^{++}$ | $2^{++}$ | $1^{+}$ | $3^{+++}$ |
| WFA | $2^{+++}$ | $2^{++}$ | $2^{+++}$ | $4^{+++}$ | $2^{++}$ | $2^{++}$ | $2^{+}$ | $3^{+++}$ |
| SNA | 0 | 0 | 0 | 0 | 0 | 0 | 0 | 0 |
| UEA-1 | 0 | 0 | 0 | 0 | 0 | 0 | 0 | 0 |
| PNA | $1^{++}$ | $3^{++}$ | $2^{++}$ | $4^{+++}$ | $1^{++}$ | $3^{+++}$ | $1^{+}$ | $3^{++}$ |

Numbers indicate staining frequency on a semiquantitatative scale ranging from 0 to 5 where 0 corresponds to no reactive cells, 1 to occasional, 2 to a few, 3 to a moderate number and 4 to numerous reactive cells. Staining intensity, (+++) strong; (++) moderate; (+) weak.

*© Cancer Research Campaign 1997*

**Figure 6** Labelling of HT29 tumour with SBA in a SCID mouse treated with 5-FU and killed at day 26. Cells identified as signet ring cells and enterocyte-like differentiated tumour cells are strongly labelled. Bar = 50 μm

**Figure 7** VVA-binding sites in a tumour of a 5-FU-treated SCID mouse at day 26. Signet ring cells (S) and enterocyte-like differentiated tumour cells (E) are heavily labelled. Bar = 50 μm

**Figure 8** HT29 tumour from a 5-FU-treated SCID mouse stained with WFA. A cluster of enterocyte-like differentiated tumour cells (arrow) and a signet ring cell (S) are strongly labelled. Bar = 50 μm

**Figure 9** PNA binding in a SCID mouse treated with 5-FU. The signet ring cell (S) and enterocyte-like differentiated tumour cells (E) are labelled. Bar = 50 μm treated with 5-FU. The other GalNAc-specific lectins (SJA and SNA) and fucose-specific lectin (UEA-I) did not react with HT29 cells in either the 5-FU-treated or the saline-injected control animals (see Table 2). There were considerable differences in the binding of PNA, a galactose-specific lectin. In HT29 tumours of 5-FU-treated mice, the signet ring cells and enterocyte-like differentiated tumour cells showed increased binding of PNA (Figure 9) compared with the controls.

### DISCUSSION

The SCID mouse model of metastatic HT29 human colon cancer established in our laboratory has recently been shown to be of clinical relevance as the glycoconjugate expression indicative of metastasis is the same in patients as it is in the human-derived tumours grown in SCID mice (Schumacher et al, 1994 *a,b*) and was used for the present study. The aim of the present study was to investigate whether 5-FU chemotherapy in this clinically relevant model system induces any change in cell number by modifying the mitotic and/or apoptotic indices of tumour cells. As apoptosis is lower than mitosis in a growing tumour, quantification of apoptotic and mitotic indices in a 5-FU-treated human colonic adenocarcinoma xenograft system may elucidate the proliferation profile of this disease. Our results show that, 24 h and 48 h after the end of the drug treatment, the transplanted tumours showed a decreased mitotic index in response to 5-FU, whereas the apoptotic index was enhanced. In principle, chemotherapeutic treatment of tumour cells can lead to an elongation of the time in which the tumour cells are in mitosis (Camplejohn et al, 1980) and, in particular, 5-FU can lead to a decrease in the mitotic index (Camplejohn et al, 1977). It has been reported that anti-cancer drugs induce apoptosis in colorectal carcinoma cells (Makino et al, 1996), and measurement of apoptotic index may be important in cancer chemotherapy (Williams, 1991; Wyllie, 1993).

Our data of tumour growth and apoptotic index indicate that the 5-FU-induced toxicity may be related in part to the enhanced apoptosis in tumour cells. However, more detailed studies that investigate the effect of 5-FU on the length at which apoptotic figures can be observed have to be performed.

In this study, the therapeutic activity of 5-FU has been measured using the tumour weight 24 h and 48 h after the end of the drug treatment as a criterion of tumour growth inhibition. Treatment of tumour xenografts with 5-FU for 4 days was found to reduce the tumour weight significantly compared with the controls. Our data therefore indicate that continuous infusion of 5-FU has significant in vivo therapeutic efficacy against this HT29 human colon cancer model. Further studies are needed to evaluate whether 5-FU treatment also has any influence on metastases of this tumour to the lungs.

© Cancer Research Campaign 1997 

The HT29 cell line is a multipotent cell population that differentiates into the phenotype of mature enterocytes (HT29-18-C1) and mucus-secreting goblet (HT29-18-N2) cells (Huet et al, 1987). In our study, the quantitative assessment of cell proliferation in HT29 tumours has shown that the nuclear antigen detected by Ki-67 is expressed more in the enterocyte-like differentiated cells than in the signet ring cells. Although the therapeutic failure could be due to re-entry of signet ring cells into cell cycle, it would be interesting to assess whether after cell division these cells differentiate into mucin-containing goblet cells or enterocyte-like tumour cells. The slow proliferation rate of the KI-67 positive goblet cells might account for the failure of chemotherapy that interferes with the cell division mechanisms. It would therefore be of interest to evaluate the number of signet ring cells in HT29 tumours of those SCID mice treated by an apoptosis-inducing drug. These drugs should therefore target both enterocyte-like and goblet cell-like tumour cells equally. In this study, the increase in Ki-67 mean labelling index 24 h after 5-FU treatment appears to be due to the prolongation of cell cycle in enterocyte-like tumour cells (Camplejohn et al, 1977). The decrease of Ki-67 labelling index 48 h after the end of 5-FU treatment was consistent with the finding of lower mitotic rate, lower tumour growth and increased apoptosis at this stage.

The PAS reaction showed appreciable differences in the goblet cell-like differentiation of signet ring cells in HT29 tumour cells of 5-FU-treated animals and saline-injected controls. In the tumours grown in SCID mice, the increase in the distribution of PAS-positive cells after 5-FU treatment shows a shift towards the signet ring cell phenotype compared with enterocyte stem cell phenotype. The possible reasons for the accumulation of signet ring tumour cells could be a differentiating effect of 5-FU or a depletion of the enterocyte stem cell phenotype by 5-FU. The latter explanation seems more likely as only very few goblet-like cells were Ki-67 positive, indicating that the majority of these cells were non-replicating and thus less sensitive to 5-FU treatment. The lectin-binding pattern also reflected an increase in the expression of particular glycoconjugates in response to 5-FU treatment. The more marked binding of SBA, VVA, WFA and PNA, particularly to the signet ring cells, would indicate that the 5-FU induces an increase of *N*-acetylgalactosamine and galactose residues to the glycoconjugate core of intracellular mucins of HT29 cells. Although the mechanisms by which anti-metabolites can affect glycosylation are not completely understood (De Graff et al, 1993), our results suggest that a 5-FU-induced increase in differentiation of mucin-producing HT29 tumour cells could be responsible for changes in their cellular glycoconjugates. However, other factors, such as alterations in cell membrane sialoglycoconjugates (Hindenburg et al, 1985) or the changes in activities of specific glycosyltransferases (Paulson and Colley, 1989), might be additional mechanisms of 5-FU in influencing cellular glycoconjugate composition.

In summary, our study demonstrates that anti-tumour activity of 5-FU is not confined to its effects on cell proliferation and apoptosis but is also apparent in glycoconjugate expression of HT29 cells implanted in SCID mice. However, the correlation between the 5-FU-induced changes in mitotic and apoptotic indices on the one hand and changes in glycosylation on the other, particularly at the level of the intermediate carbohydrate metabolism, remain to be resolved. Our data on effects of 5-FU on local tumour development may serve as a basis for further studies aimed at the identification of regulatory factors involved in the metastatic spread of tumour cells in SCID mice. This might lead to new chemotherapeutic concepts for influencing local tumour growth and metastasis of human colonic carcinoma.

### ACKNOWLEDGEMENT

The authors would like to thank Professor Dr H Korr, RWTH Aachen, for his many helpful suggestions and criticisms.

### REFERENCES

Bengmark S and Hafstrom L (1969) The natural history of primary and secondary malignant tumours of the liver: the prognosis for patients with hepatic metastases from colonic and rectal carcinoma by laparotomy. *Cancer* **23**: 198–202

Buyse M, Zeleniuch-Jacquotte A and Chalmers TC (1988) Adjuvant therapy of colorectal cancer: why we still don't know. *JAMA* **259**: 3571–3578

Camplejohn RS, Schultze B and Maurer W (1977) In vivo cell synchrony in the L1210 mouse leukaemia studied with 5-fluorouracil or 5-fluorouracil followed by cold thymidine infusion. *Br J Cancer* **35**: 546–566

Camplejohn RS, Schultze B and Maurer W (1980) An in vivo double labelling study of the subsequent fate of cells arrested in metaphase by vinchristine in the JB-1 mouse ascites tumour. *Cell Tissue Kinet* **13**: 239–250

Cunningham D and Findlay M (1991) The chemotherapy of colon cancer can no longer be ignored. *Eur J Cancer* **29**: 2077–2079

De Graff TW, Slot SS, Peters GJ and Van Dijk W (1993) Changes in glycosylation of L1210 cells after exposure to various antimetabolites. *Eur J Cancer* **29A**: 1760–1765

Hindenburg AA, Taub RN, Grant S, Chang G and Baker MA (1985) Effects of pyrimidine antagonists on sialic acid regeneration in HL-60 cells. *Cancer Res* **45**: 3048–3052

Ho SB, Yan PS, Dahiya R, Neuschwander-Tetri BA and Basbaum CKIM (1994) Stable differentiation of human colon adenocarcinoma cell line by sodium butyrate is associated with multidrug resistance. *J Cell Physiol* **160**: 213–226

Huet C, Sahuquillo-Merino C, Coudier E and Louvard D (1987) Absorptive and mucus secreting subclones isolated from a multipotential intestinal cell line (HT-29) provide new models for cell polarity and terminal differentiation. *J Cell Biol* **105**: 345–358

Imaizumi M, Kondo T, Tagichi T, Hattori T, Abe O, Kitano M and Wakui A (1993) A standardized method of using nude mice for the in vivo screening of antitumour drugs for human tumours. *Surgery Today* **23**: 412–419

Laffer UT for The SAKK (1995) Long-term results of single course adjuvant intraportal chemotherapy for colorectal cancer. *Lancet* **345**: 349–353

Makino M, Shirai H, Sugamura K, Kimura O, Maeta M, Itoh H and Kaibara N (1996) Increased induction of apoptosis of human colorectal cancer cells after preoperative treatment with 5-fluorouracil. *Oncology Reports* **3**: 281–285

Marsoni S for the Impact Investigators (1995) Efficacy of adjuvant fluorouracil and folinic acid in colon cancer. *Lancet* **345**: 939–944

Momoi T, Shinmoto M, Kasuya J, Senoo H and Suzuki Y (1986) Activation of CMP-N-acetylneuraminic acid:lactosylceramide sialyltransferase during the differentiation of HL-60 cells induced by 12-O-tetradecanonylphorbol-13-acetate. *J Biol Chem* **261**: 16270–16273

Paulson JC and Colley KJ (1989) Glycosyltransferases: structure, localization, and control of cell-type-specific glycosylation. *J Biol Chem* **264**: 17615–17618

Peters GJ, Pinedo HM, Ferwerda W, De Graff TW and Van Dijk W (1990) Do antimetabolites interfere with the glycosylation of cellular glycoconjugates? *Eur J Cancer* **26**: 516–523

Pratt WB and Palmer Taylor (1990) *Principles of Drug Action: the Basis of Pharmacology*, 3rd edn. Churchill Livingstone: New York

Schumacher U, Adam E, Flavell DA, Boehm D, Brooks SA and Leathem AJ (1994*a*) Glycosylation pattern of the human colon cancer cell line HT 29 detected by *Helix pomatia* agglutinin and other lectins in culture in primary tumours and in metastases in SCID-mice. *Clin Exp Metastas* **12**: 398–404

Schumacher U, Higgs D, Loizidou M, Taylor I and Leathem A (1994*b*) The lectin *Helix pomatia* agglutinin is a good prognostic marker in colon cancer. *Cancer* **74**: 3104–3107

Silveberg E and Lubera JA (1989) Cancer statistics 1989. *CA*, **39**: 3–20

Williams GT (1991) Programmed cell death: apoptosis and oncogenesis. *Cell* **65**: 1097–8

Wyllie AH (1992) The 1992 Frank Rose Memorial Lecture. *Br J Cancer* **67**: 205–8

© Cancer Research Campaign 1997